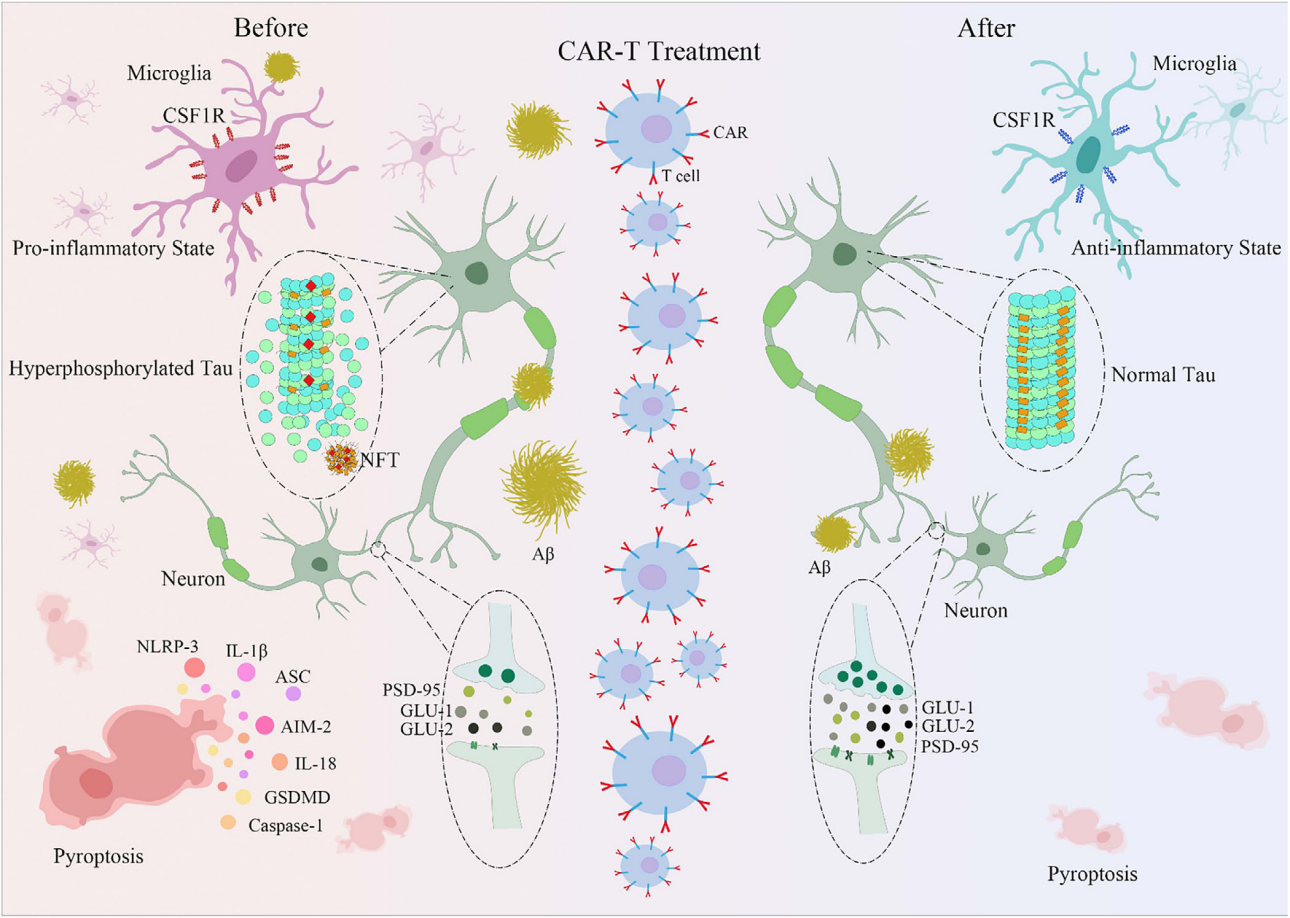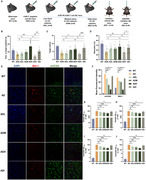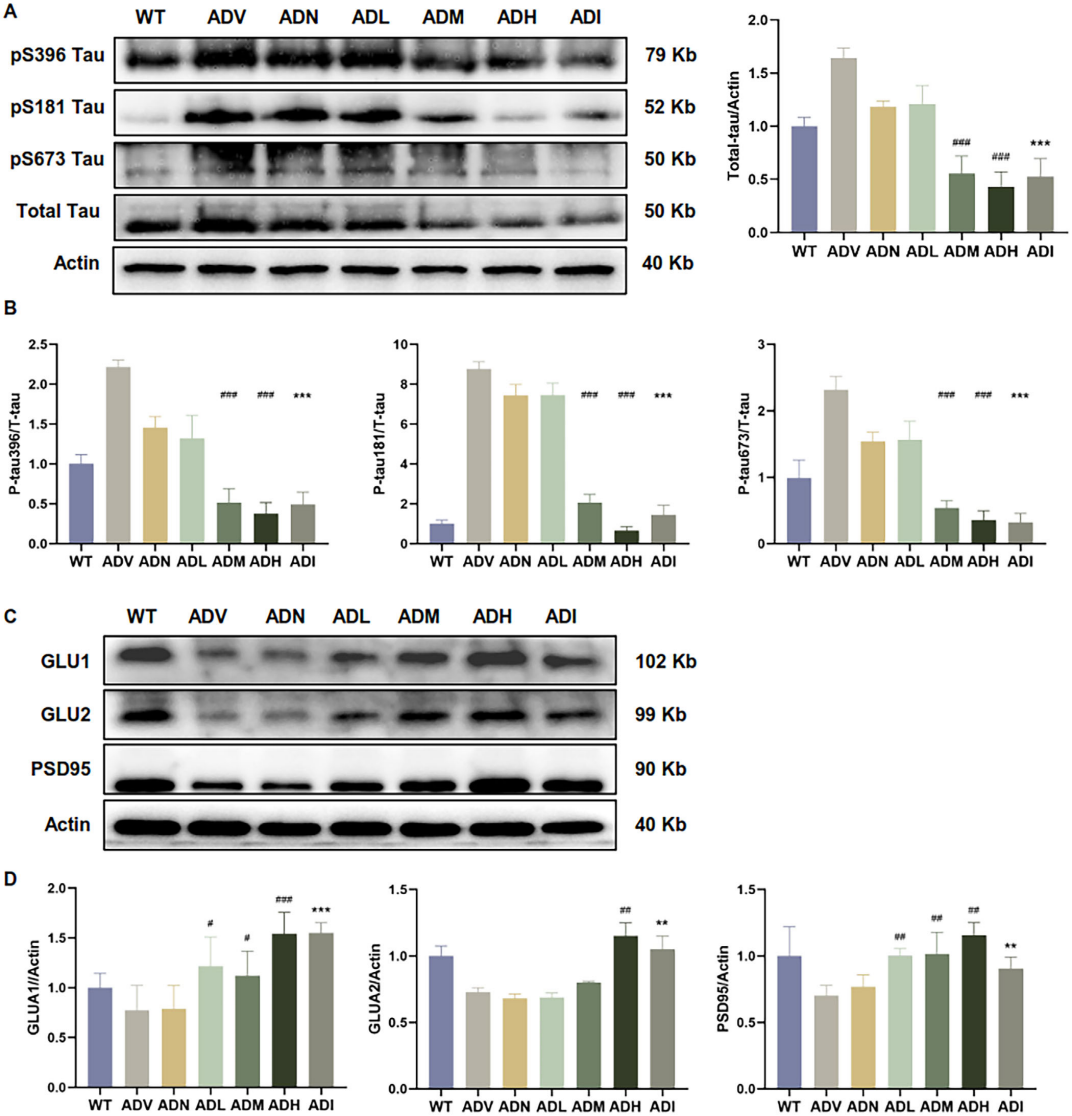# Chimeric antigen receptor T cell targeting colony‐stimulating factor 1 receptors alleviates amyloid‐β and tau pathologies in Alzheimer’s disease‐pathologic male mice

**DOI:** 10.1002/alz.094758

**Published:** 2025-01-09

**Authors:** Jun Xu, Yanli Wang, Feng Yu, Xiaohong Wang, Jiwei Jiang, Tianlin Jiang

**Affiliations:** ^1^ Beijing Tiantan Hospital, Capital Medical University, Beijing China; ^2^ School of Public Health, Shandong Second Medical University, Weifang, Shandong China; ^3^ School of Life Sciences, Jiangsu University, Zhenjiang, Jiangsu China; ^4^ Institute of Translational Medicine, Medical College, Yangzhou University, Yangzhou, Jiangsu China; ^5^ Beijing Tiantan Hospital, Capital Medical University, Beijing China; ^6^ Department of Neurology, Beijing Tiantan Hospital, Capital Medical University, Beijing, Beijing China

## Abstract

**Background:**

Microglia play a critical role in the pathogenesis and development of Alzheimer’s disease (AD). Selective small‐molecule colony‐stimulating factor 1 receptors (CSF1R) inhibitor, designed to deplete microglia, could be used to meliorate AD. This study aimed to investigate the effects and mechanisms of chimeric antigen receptor T (CAR‐T) cells targeting CSF1R in 6‐month‐old APP/PS1 male mice.

**Method:**

The Morris water maze test, Y‐maze test, tail suspension test, swimming test, nesting score assay, and three‐chamber social interaction assay were performed to assess cognitive function. Western blotting, immunofluorescence staining, enzyme‐linked immunosorbent assay, and Bio‐Plex Pro Mouse Cytokine 23‐plex assays were conducted to explore AD‐core pathologies, neuroinflammation, microglial activation and polarization, and pyroptosis.

**Result:**

iCSF1R.CAR‐T cells ameliorated cognitive deficits and reduced β‐amyloid load, tau hyperphosphorylation, and synapse‐related proteins, as well as microglial activation and polarization in 6‐month‐old APP/PS1 male mice. Furthermore, iCSF1R.CAR‐T cells significantly inhibited pyroptosis and attenuated neuroinflammation in these mice.

**Conclusion:**

The results suggest that iCSF1R.CAR‐T cells improve cognitive decline and neuroinflammation, possibly by suppressing caspase‐1/GSDMD‐mediated pyroptosis. Consequently, iCSF1R.CAR‐T cells could be a potential therapeutic approach for AD and other neurological disorders characterized by microglial dysfunction.